# The effect of biological sealants and adhesive treatments on matrix metalloproteinase expression during renal injury healing

**DOI:** 10.1371/journal.pone.0177665

**Published:** 2017-05-11

**Authors:** José Miguel Lloris-Carsí, Carlos Barrios, Beatriz Prieto-Moure, José Miguel Lloris-Cejalvo, Dolores Cejalvo-Lapeña

**Affiliations:** 1 Department of Surgery, University of Valencia, Valencia, Spain; 2 Division of Experimental Surgery, Valencia Catholic University, Valencia, Spain; Kyoto Daigaku, JAPAN

## Abstract

**Background:**

Renal injuries are relatively common in cases of abdominal trauma. Adhesives and sealants can be used to repair and preserve damaged organs. Using a rat model, this study explores the activity of different matrix metalloproteinases (MMP) during the healing of renal injuries treated by two biological adhesives (TachoSil and GelitaSpon) and a new synthetic elastic cyanoacrylate (Adhflex).

**Methods:**

Renal traumatic injuries were experimentally induced in 90 male Wistar rats by a Stiefel Biopsy Punch in the anterior aspect of the left kidney. Animals were divided into five groups: 1, sham non-injured (n = 3); 2, non-treated standard punch injury (n = 6); 3, punch injury treated with TachoSil (n = 27); 4, punch injury treated with GelitaSpon (n = 27); and, 5, punch injury treated with Adhflex (n = 27). Wound healing was evaluated 2, 6, and 18 days after injury by determining the expression of MMPs, and the histopathological evolution of lesions.

**Findings:**

Histologically, the wound size at 6 days post-injury was larger in Adhflex-treated samples than in the other treatments, but the scarring tissue was similar at 18 days post-injury. Only the MMPs subtypes 1, 2, 8, 9, and 13 were sufficiently expressed to be quantifiable. Both time since injury and treatment type had a significant influence on MMPs expression. Two days after injury, the expression of MMP8 and MMP9 was predominant. MMP2 expression was greater 6 days after injury. The Adhflex-treated group had a significantly higher MMPs expression than the other treatment groups at all healing stages.

**Conclusions:**

All three sealant treatments induced almost similar expression of MMPs than untreated animals indicating a physiological healing process. Given that all renal trauma injuries must be considered emergencies, both biological and synthetic adhesives, such as Adhflex, should be considered as a treatment options.

## Introduction

Penetrating abdominal trauma is a major reason for emergency admission in The USA [1]. The use of biological adhesives has been attracting growing interest since 1998, when The Food and Drug Administration (FDA) approved Dermabond, a cyanoacrylate, for clinical use including the application of biological adhesives in cases of renal injuries [2] and biopsies (e.g., traumatic renal lesion and laparoscopic lesion in polycystic kidney disease) [3,4].

Matrix metalloproteinases (MMPs) are enzymes with metal ion-dependent activity that degrade extracellular matrix (ECM) glycoproteins. MMPs play a crucial role in various biological processes, such as embryogenesis, tissue remodeling, angiogenesis, and wound healing [5]. During the past two decades the role of MMPs in the morphogenesis of various organs, including that of the metanephros, has been investigated extensively. Mammalian nephrogenesis comprises a series of intricate events characterized by a sustained remodeling and turnover of ECM, suggesting a potential role of MMPs in renal development. [5]

The normal remodeling and healing is a result of the balance between extracellular matrix protein synthesis and degradation. [6] At the initial phase of healing, MMPs are activated by inflammatory cells such as macrophages or neutrophils. MMPs can also be activated by damaged epithelial cells, or the stroma of the repairing tissues. [7,8] MMPs participate in the regulation of cytokine activity, either by direct cell proteolysis or by direct activation or inhibition of cytokines. In cases such as CXCL8 or CXCL5, its activation by MMPs produces a potentiation of the activity of other cytokines, which finally results in an increase in the recruitment of inflammatory cells in the injured area. [9]

During the healing of tissue injuries, MMPs play a major role in degrading the components of the ECM, such as collagen or fibrin, to facilitate cell migration, deposition of new components in the ECM, and the development of regenerating tissue. [10] Throughout wound healing, MMP activity is regulated by a combination of transcriptional control (e.g., by Interleukin-1 and Tumor necrosis factor alpha), the presence or absence of factors required to transform proenzymes into their active forms, and the direct activity of MMPs inhibitors (TIMPs).[11,12,13]

Upregulation of MMP-2 and MMP-9 is found in acute kidney injury. MMP-2 and MMP-9 are implicated in the degradation of the tight junction protein zonula occludens-1 in the glomerulus [14], MMP-9 is considered to contribute to the degradation of occluding endothelial cells, resulting in increased vascular permeability. [15] These data suggest that MMP mediate acute kidney injury and play a role in the changes of the vascular endothelium, glomeruli and tubular epitelial cells. Adhesion molecules have also been identified as critical targets of MMP in the kidney, consistent with the increased vascular and tubular permeability characteristic of acute kidney injury. [16] Therefore, the clinical utility of measuring MMPs expression during the healing course of penetrating renal injuries could provide new insights about the repair process of these lesions. [17]

Clarification of the renal repair process is crucial for developing novel therapeutic strategies for kidney injury. Thus, the purpose of this study was first to test if the use of different sealants, two biological adhesives (TachoSil and GelitaSpon) and a synthetic elastic cyanoacrylate (Adhflex), could interfere the normal repairing histologic outcome of renal injuries in a rat model. This study was also aimed at exploring the activity of MMPs during the healing of renal injuries treated with the three procedures. Beyond histology, MMPs expression could reflect the different biochemical response to sealants during the healing process. Kidney injuries were made on the anterior aspect using a Stiefel Biopsy Punch, and were locally repaired by the three mentioned sealants. Histopathological changes and MMP activities were monitored during renal injury healing, at 2, 6 and 18 days post-injury. A non-treated injured group was also included.

## Methods

The experimental design and animal welfare procedures were approved by the Animal Welfare Committee (Research Ethical Committee for Animals Studies) of the Valencia Regional Government (ref. number: 2015/VSC/PEA/00097) in compliance with applicable legislation (Royal Decree 53/2013), and FDA recommendations on animal welfare in experimentation.

### Animals

Ninety male Wistar rats (300–350 g) (Harlan Laboratories) were kept in a standard animal facility, with access to food and water both pre-operatively and postoperatively. Animals surveillance and care was done every 12 hours in the preoperative period and every 6 hours throughout the whole postoperative process (18 Days). No animal become severely ill or died during the 18 days of the experiment.

### Experimental groups

Animals were divided into five groups: 1: sham non-injured (n = 3); 2: non-treated (n = 6); 3: TachoSil treated (n = 27); 4: GelitaSpon treated (n = 27), and 5: Adhflex treated (n = 27). En each treatment group, 5 rats were use for histological studies and 4 for MMP expression at the 3 stages of the study: 2, 6 and 18 days post–injury (9x3 = 27rats). Two of the untreated rats were used for both histology and MMPs expression in each stage of the study (2X3 = 6). In the control rats, samples were divided in two portions to have at least two histological studies and two MMPs determinations in each experimental period.

### Biomaterials and adhesive used

Adhflex® (*Bioadhesives Medtech Solutions s*.*l*., *Alicante*, *Spain*) is a cyanoacrylate-based adhesive supplemented with acrylates to enhance elasticity, reduce stiffness and increase cohesive strength. Adhflex® has a greater polymer flexibility and lower polymerization temperature than other clinically used cyanoacrylates. TachoSil® (*Takeda GmbH-Austria*) is a haemostatic sponge containing human fibrinogen (5.5 mg per cm^2^) and human thrombin (2.0 IU per cm^2^). GelitaSpon®(*Gelita MedicalGmbH-Germany*) is a biodegradable, topical haemostat (absorbable, oxidized cellulose sponge). All three products have adhesive and coagulant properties.

### Anaesthesia

Animals were intraperitoneally anaesthetized with Ketamine (80 mg kg^-1^) and Xylazine (10 mg kg^-1^), spontaneous breathing being maintained during a midline abdominal laparotomy in which the punch injury was created. Partial O_2_ and CO_2_ pressure was monitored trans-cutaneously, ensuring optimal haematosis throughout the surgical procedure. To minimize pain, animals were treated with Buprenorphine (0,1 mg/kg every 12 h) the first 48 hours after surgery.

### Standardized treatments

Once the anaesthesia was completed, the abdominal cavity was exposed via a midline incision. Throughout a transperitoneal approach, the right kidney was located and carefully exposed by retracting the bowel loops. With direct vision, renal injuries were performed on the anterior face of the kidney by using a Stiefel biopsy punch. Lesions homogeneous in size (8 mm diameter) and depth (4 mm) were easily obtained by applying a small pressure over the surface together with a slight twist. In the TachoSil® and GelitaSpon® groups, the wounds were covered with homogeneous circles to the biological adhesives. In the Adhflex group, a single drop (21.3 ± 1.2 mg) was applied to each wound using the supplied applicator. After sealing the renal injury, the kidneys were observed for a further 3 min to ensure hemorrhagic occlusion. Finally, 1 ml saline at 37°C was injected into the abdomen and the incision was sutured in two layers.

### Euthanasia

Nine animals from each of the treatment groups were euthanized by intraperitoneal injection of sodium pentobarbital (lethal dose, 60 mg/kg) at 2, 6 and 18 days post–injury, and samples were processed for histology and MMPs determinations.

### Healing evaluation

#### 1) Metalioproteinases expression

During healing, the activation and inhibition of different MMPs affects multiple processes. To test whether TachoSil®, GelitaSpon® or Adhflex® stimulated the local secretion of MMPs by the host kidney cells, kidney homogenates (60 μl of serum) we subjected to enzyme-linked immunosorbent assay (ELISA), testing a panel of MMPs (Mosaic ELISA MMP Panel, *R and D Systems*).

MMPs were quantified by chemiluminescence (according to the manufacturer’s instructions). MMP-1, MMP-2, MMP-3, MMP-7, MMP-8, MMP-9 and MMP-13 levels were quantified. The panel and protocol have a sensitivity in the pg/ml range. The MMP levels were quantified by gel densitometry (Image J), using the mean of duplicate samples. Equal spot sizes were analysed per blot.

The Mosaic ELISA MMP Panel can detect up to seven different MMPs. However, because of limits of sensitivity, we restricted our analyses to only the most highly expressed MMPs.

#### 2) Histological studies

Haematoxylin-Eosin staining (3 µm thick slices) was used for the overall study of the samples. Specific stains were used to observe histological changes produced in the development of renal lesions and the consequences of several treatments. Five kidney tissue samples from each group were examined. Gap between lesion was measured in μm.

#### Statistical analysis

SPSS Statistics (version 20.0, IBM, Armonk, NY, USA) was used for all statistical analyses. Fisher’s Least Significant Difference (LSD) test, were used to identify significant between group differences (p<0.05) in the size of the gap between wound edges. Due to the small sample size, the non parametric Kruskal-Wallis test was used to compare differences in MMPS expression among the groups. Mann-Whitney U test was used to analyze differences in MMPs expression between the untreated and each treated group. *P*-values less than 0.05 were considered statistically significant.

## Results

### Matrix metalloproteinases expression

**Fig 1:**
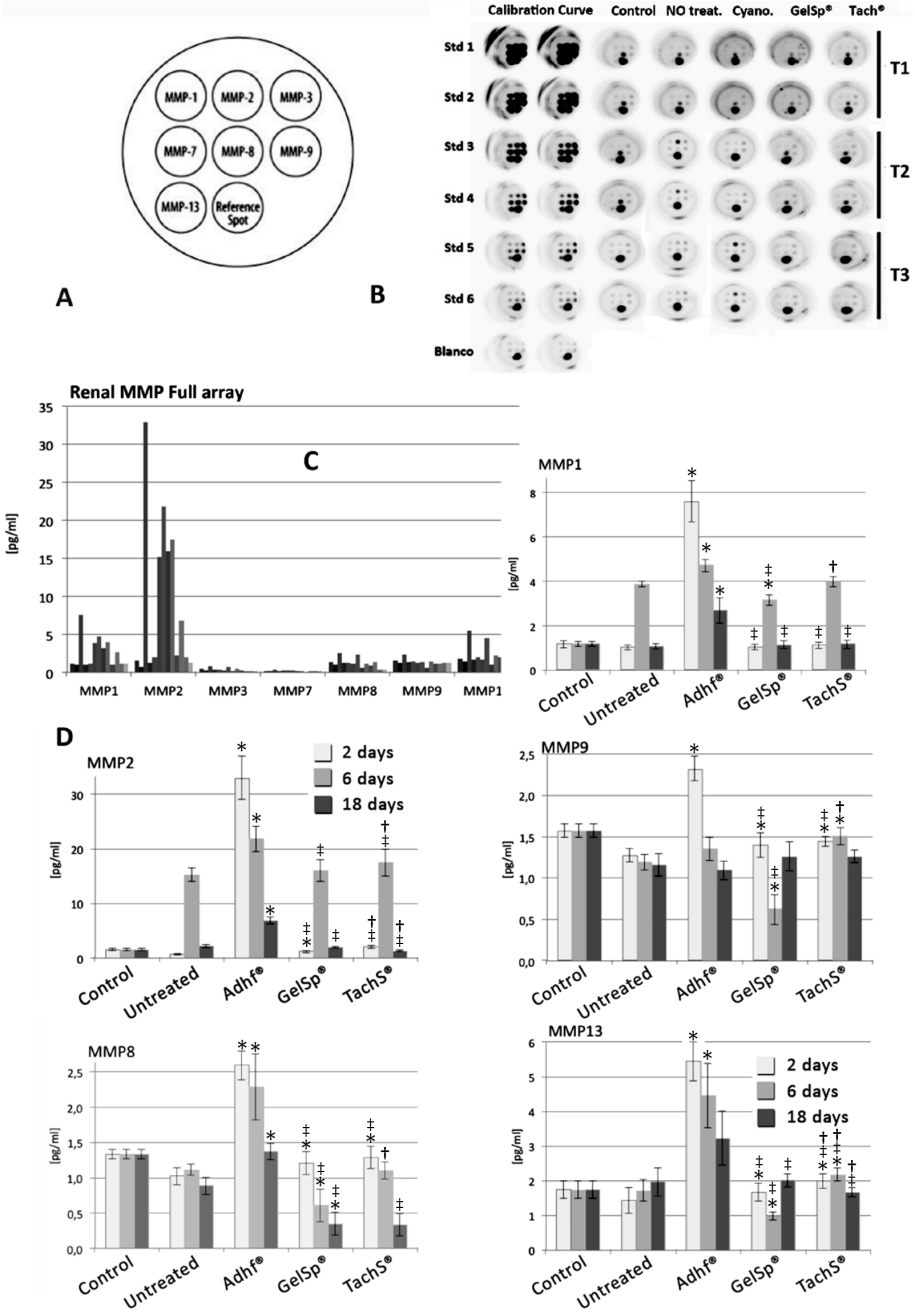
Metalloproteinases expression in sham-control, non-treated and treated (Adhflex, GelitaSpon and TachoSil) kidneys. (A) Chart showing the most expressive metalloproteinase; (B) ELISA MMP panel (*R&D Systems*) plates of the rat metalloproteinase in all groups; (C) Individual graphs for the most expressed metalloproteinases in all groups (control, non-treated, and Adhflex, GelitaSpon and TachoSil treated) tested at 2, 6 and 18 days post-injury. Mann-Whitney U test: (*), p<0.05 as compared to untreated animals; (‡), p<0.05 as compared to Adhf® group; (†), p<0.05 as compared to GelSp® group. Kruskal-Wallis test shows the statistical differences among four groups in each healing time period. (^ns^): no significance.

Of the MMPs included in the Mosaic ELISA MMP Panel, only MMP1, MMP2, MMP8, MMP9 and MMP13 were sufficiently expressed to be quantifiable (**Fig 1**) (**Table 1**).

**Table 1:** MMP1, MMP2, MMP8, MMP9 and MMP13 Metalloproteinase values (pg/mL) in the three healing times (T1: 2 days after injury; T2: 6 days, and T3: 18 days) and in each treatment group.

	Time	Untreated (n = 4)	Adhf® (n = 4)	GelSp® (n = 4)	TachS® (n = 4)	Kruskal-Wallis test (p value)
MMP 1	**T1**	1,01 ± 0,07	7,61±0,13*	1,01 ± 0,12‡	1,11 ± 0,04‡	0.015
	**T2**	3,87 ± 0,11	4,71 ± 0,17*	3,15 ± 0,21*‡	3,99 ± 0,12‡†	0.004
	**T3**	1,05 ± 0,08	2,69 ± 0,55*	1,13 ± 0,06‡	1,67 ± 0,07‡	0.019
	**Kruskal-Wallis test (**p value)	0.022	0.007	0.018	0.021	
MMP 2	**T1**	0,66 ± 0,10	33,10 ± 0,10*	1,19 ± 0,02*‡	2,03 ± 0,07*‡†	0.003
	**T2**	15,23 ± 0,18	21,82 ± 0,25*	15,98 ± 0,93‡	17,51 ± 0,42*‡†	0.005
	**T3**	2,19 ± 0,13	6,83 ± 0,27*	1,97 ± 0,19‡	1,29 ± 0,12*‡†	0.004
	**Kruskal-Wallis test (**p value)	0.007	0.007	0.007	0.007	
MMP 8	**T1**	1,02 ± 0,11	2,59 ± 0,1*	1,20 ± 0,05*‡	1,28 ± 0,05*‡	0.004
	**T2**	1,11 ± 0,07	2,28 ± 0,46*	0,60 ± 0,22*‡	1,09 ± 0,11‡†	0.005
	**T3**	0,88 ± 0,11	1,36 ± 0,11*	0,34 ± 0,16*‡	0,33 ± 0,15*‡	0.005
	**Kruskal-Wallis test (**p value)	0.069^ns^	0.018	0.012	0.007	
MMP 9	**T1**	1,27 ± 0.80	2,32 ± 0,05*	1,39 ± 0,05*‡	1,44 ± 0,06*‡	0.006
	**T2**	1,18 ± 0,09	1,35 ± 0,13	0,62 ± 0,17*‡	1,50 ± 0,10*†	0.006
	**T3**	1,15 ± 0,13	1,09 ± 0,10	1,26 ± 0,17	1,25 ± 0,07	0.208^ns^
	**Kruskal-Wallis test (**p value)	0.427^ns^	0.010	0.015	0.019	
MMP 13	**T1**	1,42 ± 0,06	5,44 ± 0,06*	1,65 ± 0,06*‡	1,99 ± 0,10*‡†	0.003
	**T2**	1,72 ± 0,30	4,46 ± 0,91*	0,98 ± 0,10*‡	2,18 ± 0,17*‡†	0.003
	**T3**	1,96 ± 0,39	3,22 ± 0,77	2,01 ± 0,08‡	1,66 ± 0,14‡†	0.014
	**Kruskal-Wallis test (**p value)	0.107^ns^	0.037	0.007	0.014	

Mann-Whitney U test: (*), p<0.05 as compared to untreated animals; (‡), p<0.05 as compared to Adhf® group; (†), p<0.05 as compared to GelSp® group. Kruskal-Wallis test shows the statistical differences among four groups in each healing time period. (^ns^): no significance.

#### MMP1

Both time since injury and treatment method, were found to influence MMP1 expression (Kruskal-Wallis test for time, p< 0.05; for treatment, p<0.05). In the untreated and in all treated groups differences in the MMP1 expression were detected between 2, 6 and 18 days post-injury. (Table 1). The highest mean MMP1 expression level was recorded at 6 days post-injury In all groups. The lowest mean MMP1 concentration was recorded at 18 days post-injury. No differences were found in MMP1 concentration between the non-treated and either the TachoSil or GelitaSpon-treated groups. The Adhflex-treated group had the highest MMP1 concentration, which was significantly higher than for the other treatments. (Table 1).

#### MMP2

Similarly than in MMP1, both time since injury and treatment influence MMP2 expression. There were significant differences in MMP2 expression between the 2, 6 and 18 days post-injury sampling times in all groups, being more pronounced in the Adhflex-treated group (Kruskal-Wallis test, p<0.01) (Table 1). MMP2 expression was also greatest at 6 days post-injury and the lowest at 18 days post-injury (Table 1)(Fig 1). No differences in MMP2 expression were detected between the non-treated and GelitaSpon-treated groups at 6 and 18 days after injury. In both cases, MMP2 showed the lowest mean concentration recorded (Table 1). The Adhflex-treated group had the highest expression of MMP2 (Fig 1).

#### MMP8

Significant differences in MMP8 expression between the 2, 6 and 18 days post-injury were detected in the three treated groups (p<0.01), but not in the untreated group (Table 1). MMP8 expression decrease as healing time progressed (Fig 1). There were differences between GelSp and TachS groups only at T2 sampling time (Table 1). MMP8 expression was also greatest at 2 days post-imjury. The MMP8 concentration was lowest in the GelitaSpon and TachoSil-treated groups, being significantly lower than the non-treated group. The Adhflex-treated group had a significantly higher MMP8 concentration than the GelitaSpon and TachoSil-treated groups (Table 1)(Fig 1).

#### MMP9

Almost similar expression pattern than that found for MMP8 was observe for MMP9. Time since injury influenced MMP9 expression in all treated groups but no in untreated animals (Table 1). At T3 time period, there were no differences among the treated and the untreated groups. MMP9 expression was lower in the GelitaSpon-treated group than in the non-treated. As in the other MMPs, MMP9 expression was greatest in the Adhflex-treated group, and this was significantly greater than for the other treatments (Table 1)(Fig 1).

#### MMP13

The expression of MMP13 followed a pattern similar to MMP8 and MMP9. Time since injury influenced MMP9 expression in all treated groups but no in untreated animals (Table 1). MMP13 decreased as healing time progressed and was significantly lower at 18 days post-injury than at 2 days post-injury. There were significant differences in MMP13 expression between the untreated group and all three treated groups. The Adhflex-treated group had the greatest MMP13 expression level, which was significantly greater than for each of the other groups (Table 1)(Fig 1).

#### Histological study

**Fig 2:**
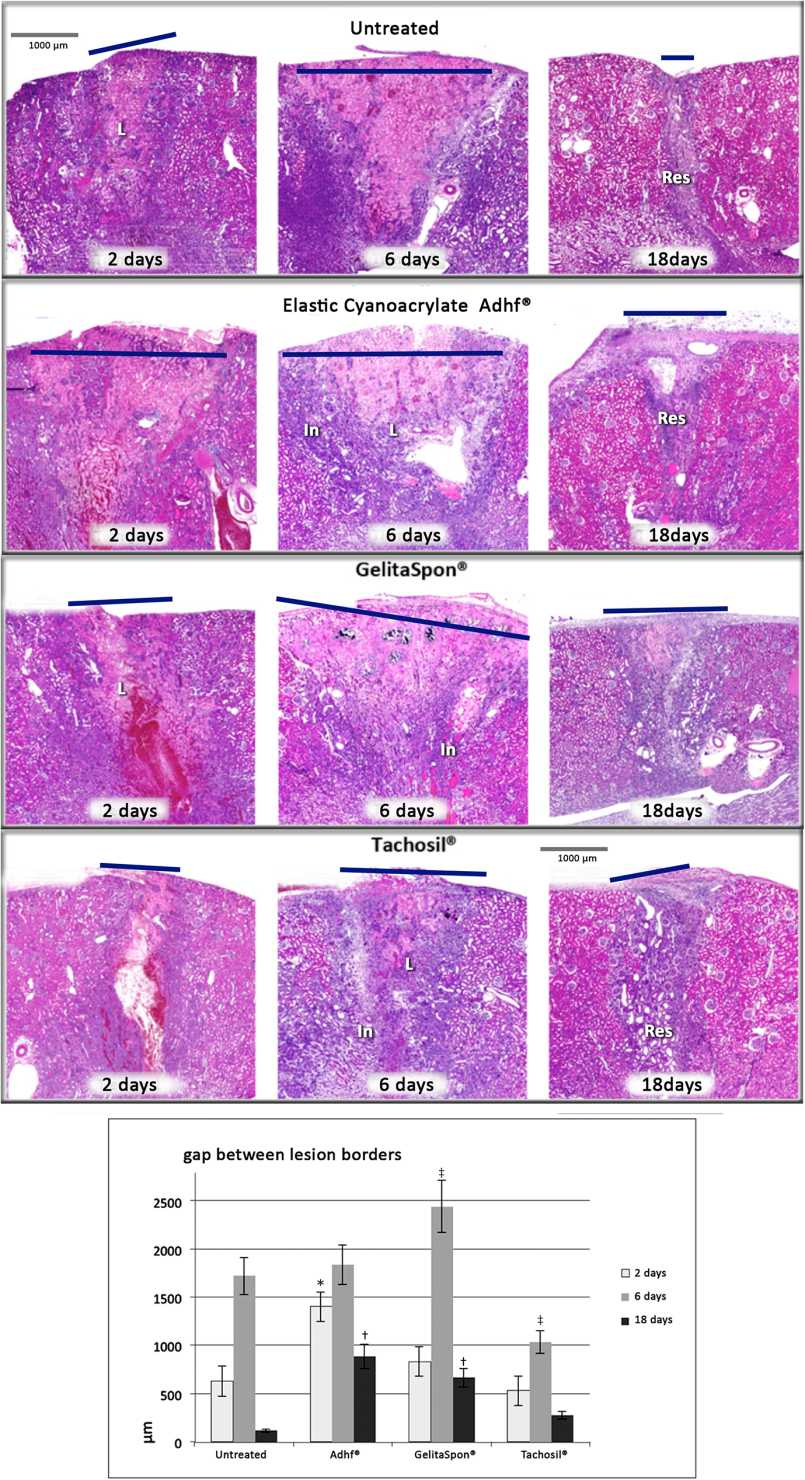
Kidney histopathology panel (Haematoxylin & Eosin) for the different treatments at 2, 6 and 18 days post-injury. Gap length between injury edges is marked with a dark line. (L) Punch lesion (In) Perilesional inflammatory area, (Res) Residual scar. The chart indicates measurements in μm of the gap between lesion edges. (*) p<0.05 as compared to untreated animals (2 days); (‡), p<0.05 as compared to untreated animals (6 days); (†), p<0.05 as compared untreated animals (18 days).

Fig 2 shows tissue sections stained with hematoxylin and eosin. Section of non-treated injured tissue at 18 days post-injury, showing a dark strip compatible with granulation tissue, comprising inflammatory cells and fibroblasts. Necrotic tissue had completely disappeared at 18 days post-injury and wound edges were fully aligned. A column of dark violet connective tissue could be observed, corresponding to the injury scar. In the renal capsule, a contraction of the connective tissue could be detected, indicating collagen maturation. The appearance of the parenchyma surrounding the scar was normal. Gap measurements between lesion borders are indicated and plotted in Fig 2.

In Adhflex-treated samples, the wound size at 6 days post-injury was larger than in the other treatments, and granulation tissue was observed around the coagulation necrosis. In some samples, the injury was covered by the renal capsule and perirenal fat. The TachoSil-treated wounds did not increase in size, as would be expected during the inflammatory process, although this did occur with the other treatments. At 18 days post-injury, the edges of the wound were completely aligned, and the necrotic tissue had been fully phagocytized. In the area around the scar, the renal parenchyma appeared completely normal. In the TachoSil-treated samples, the excess material had adhered to the organ surface; however, this did not seem to interfere with regeneration of the area (Fig 2).

## Discussion

Using an experimental rat model, this paper describes the evaluation of MMPs expression after application of three surgical sealants (TachoSil, GelitaSpon, Adhflex) in penetrating renal injury. The histological healing process was almost similar in response to the three biomaterial treatments. Adhflex, a novel elastic cyanoacrylate, showed comparable injury repair to those of the other conventional treatments tested. However, MMPs expression during the healing process was different in the three groups. In renal wounds treated with Adhflex, the different MMPs analyzed exhibit the highest expression at the different experimental periods evaluated. This finding indicate that a high MMPs expression as seen in the Adhflex treated group did not influence the treatment outcome since the three biomaterials exhibit similar histological repair.

The highest MMPs expression detected after Adhflex treatment could be related to the degradation products in form of formaldehyde and alkyl-cyanoacetate that Adhflex generates after applying in vivo [18]. This MMPs overexpression found at early healing stages does not seem to influence the final renal injury repair. TachoSil, a equine collagen matrix coated with fibrinogen and thrombin, is metabolized in the same way as endogenous fibrin by fibrinolysis and phagocytosis. GelitaSpon is a 100% purified gelatin on porcine collagen protein with fast and complete resorption following the physiologic process of collagen degradation. The degradation process of both TachoSil and GelitaSpon does not entails generation of non-physiologic toxic products. However, the superior adhesiveness and clotting speed showed by Adhflex in previous studies as compared to conventional treatments, suggest that this new elastic cyanoacrylate should be considered a useful sealant substance [19,20].

MMPs play key roles during the healing process, especially during the inflammatory and proliferative phases. Therefore, the sampling times used here were stablished according to the inflammatory, proliferative and maturation phases of injury healing.[21] Most MMPs act simultaneously, at times even sharing the same substrates, with the activity of one MMP often leading to the activation of others. For this reason, here we drew comparisons between groups of MMPs organized into defined subfamilies.[22]

Of the MMPs tested, only the collagenases (MMP1, MMP8 and MMP13) are capable of breaking the fibrillar collagen triple helix. Here we found that the collagenases had differing expression profiles. MMP1 expression was higher in the treated groups than in the non-treated group. The highest MMP1 expression was recorded for the Adhflex-treated group at 6 days post-injury. [23]

When an injury becomes chronic, as in dermal ulcers caused by burns, MMP1 concentration remains high after the first week of healing [24]. In chronic skin ulcers, prolonged MMP1 activity can have a critical effect on the re-epithelialization of tissues [25]. The MMP1 expression profiles described here are consistent with normal healing and was not affected by the sealant treatment type.

MMP8 expression peaked at 2 days post-injury, and was highest in the Adhflex-treated group. The MMP8 expression profiles described here are consistent with a normal healing process; MMP8 is stored in the granules of neutrophils, is released during the first few hours after injury, and its activity can be extended to the end of the inflammatory phase. [26,27]

MMP13 expression was highest at 2 and 6 days post-injury and was greatest in the Adhflex-treated group. Several studies have indicated that MMP13 activity during the early stages of healing is beneficial and related to the formation of the three-dimensional collagen matrix, as well as modification of fibroblast morphology and viability. [28,29] MMP13 also acts on the activity of myofibroblasts and angiogenesis, particularly during the formation of granulation tissue. [30]

Despite these beneficial effects, high MMP13 expression has also been documented in numerous chronic skin disorders, as well as in other chronic diseases, such as rheumatoid osteoarthritis, where it leads to a destruction of the collagen matrix.

Out of a number of matrix-degrading enzymes, gelatinases, MMP-2 and MMP-9, have been shown to play an important role in the formation and maturation of granulation tissue during wound healing. [31] MMP-2 expression is important during the prolonged remodelling phase, whereas the gelatinolytic activity of MMP-9 was demonstrated only in early wound healing, and the MMP-9 gene is upregulated when the granulation tissue matures.[31] This is in accordance to the findings of the current study. MMP2 expression was highest in the Adhflex-treated group at 6 days post-injury.

In addition, MMP2 has been shown to retard fibroblast differentiation during healing. [32] Therefore, the control of MMP2 activity could act as a means of preventing hypertrophic scarring. Various authors have described increased gelatinases expression following traumatic injury. [29,30] Nessler [33] measured MMP2 expression levels in patients with healing wounds (1, 7 and 25 days post-injury), with the highest levels detected at 7 days post-injury, which is consistent with other studies, which typically find that MMP expression peaks at between 5 and 7 days post-injury, which coincides with the completion of the inflammatory phase and the formation of granulation tissue.[34]

MMP-9 is produced by neutrophils and macrophages after different stimuli. [35] In addition, glomerular epithelial cells, mesangial cells, tubular cells, and fibroblasts have been shown to produce MMP-9 after stimulation by cytokines and growth factors.[9] In the current experimental study the expression of the MMP9 gelatinase was significantly higher at 2 days post-injury, being the adhesive Adhflex the treatment that disclosed higher concentration. There is as yet no clear consensus on the timing of peak MMP9 expression during healing.

In serum samples collected from patients with multiple trauma, MMP9 expression was significantly higher at the time of hospitalization than at 6 hours post-injury. [36] In contrast, the concentration of gelatinases in healing lesions in the oral mucosa showed two concentration peaks, occurring at 2 and 4 days post-injury.[37] The latter authors argued that keratinocyte migration might occur during the inflammatory reaction, resulting in the subsequent release of MMP9.

Our finding that MMP9 expression peaks at between 2 and 6 days post-injury may be associated with normal healing process. The Adhflex-treated group had the highest MMPs concentration. Although Adhflex induced greater expression of proteolytic enzymes, this cannot be considered as a negative effect, unless the proteolytic activity was not prolonged beyond the proliferative healing phase.

In our histopathology analyses, healing progression was similar across all of the study groups. Application of each of the sealants produced a marked coagulated hematoma at the affected area. Over time, granulation tissue could be observed around the necrotic area, with widely dispersed inflammatory infiltration.

The scar tissue initially covers a relatively large surface area in the days following the lesion although after a time (18 days) the scar area was significantly reduced (Fig 2). At 18 days post-injury, the necrotic tissue had been eliminated in almost all samples, being replaced by a strip of connective tissue, forming a scar and showing signs of contraction that indicate maturation of the scarred tissue. Healing progress was similar, regardless of the sealant used.

The total gap between the wound edges was greatest in the Adhflex-treated group at 2 and 18 days post-injury, and in the GelitaSpon-treated group at 6 days post-injury. However, the difference between the Adhflex-treated and the other treatment groups at 18 days post-injury was small, and did not seem to be significant with regard to the final scar outcome.

A large number of previous studies have postulated the benefits of using biological adhesives in urology. [38,39] Some authors reported the benefits of using cyanoacrylate as a sealing method in biopsy injuries prior to kidney transplantation. In a study including 194 renal transplants, none showed active bleeding at the biopsied site after sealing with cyanoacrylate. [4] In addition, this technique had a high rate of success, avoiding the parenchymal laceration and bleeding that are typical after traditional wound closing procedures using absorbable sutures.

The elastic capacity of the novel cyanoacrylate Adhflex is advantageous, and in the treatment of skin wounds would enable the adhesive to adapt to the dynamics and flexibility of the skin. In renal injuries, the renal surface is likely to be better protected if the adhesive being used is elastic, as is the case with Adhflex. In a previous experimental study on renal injuries, a significant reduction in bleeding time has been described using Adhflex as compared to other biological sealants [19]. Additionally, a peritoneal perilesional inflammatory reaction was observed, preventing possible bleeding recurrences in the early stages of healing [19]. Therefore, considering renal trauma injuries as an emergency, any rapid sealant, such as Adhflex, should be considered as a treatment option [19, 20].

According to the 2016 EAU guidelines [40], penetrating renal injury can be treated conservatively in selected stable patients is associated with a successful outcome in approximately 50% of stab wounds and up to 40% of gunshot wounds. Minor bleeding can be managed by the conservative treatment. These data suggest that there is still enough room for endoscopic exploration of grade 3–4 renal penetrating injuries, in patients with hemodynamic instability upon admission, in cases with continuous loss of hemoglobin during an initial observation period, and in those cases with associated organ injuries. In our opinion, the rapid application of sealants in the emergency operating room could aid to stabilize a not insignificant number of cases, avoiding major surgeries. In some cases, wound surface sealing can be an alternative to angioembolization in stable patients. At present, embolization has an important role in the non-operative management of renal trauma in hemodynamically stable patients. [41,42] However, currently there are no validated criteria to identify patients who require angioembolisation and its use in renal trauma remains heterogeneous.

## Conclusions

After analyzing all the results, it can be emphasized that all treatments used in this experimental model (TachoSil, GelitaSpon and Adhflex) induced a similar response along the healing period. MMPs levels evolved to baseline status as it seems to indicate the results of the controls group at 18 days of healing. Given that all renal trauma injuries must be considered emergencies, both biological and synthetic adhesives, such as Adhflex, should be considered as a treatment option.
